# Is Weight-Bearing Asymmetry Associated with Postural Instability after Stroke? A Systematic Review

**DOI:** 10.1155/2013/692137

**Published:** 2013-04-28

**Authors:** Jip F. Kamphuis, Digna de Kam, Alexander C. H. Geurts, Vivian Weerdesteyn

**Affiliations:** ^1^ViaReva, Centre for Rehabilitation, P.O. Box 812, 7301 BB Apeldoorn, The Netherlands; ^2^Radboud University Medical Centre, Nijmegen Centre for Evidence Based Practice, Department of Rehabilitation, P.O. Box 9101, 6500 HB Nijmegen, The Netherlands; ^3^Sint Maartenskliniek, Research, Development & Education, P.O. Box 9011, 6500 GM Nijmegen, The Netherlands

## Abstract

*Introduction*. Improvement of postural stability is an important goal during poststroke rehabilitation. Since weight-bearing asymmetry (WBA) towards the nonparetic leg is common, training of weight-bearing symmetry has been a major focus in post-stroke balance rehabilitation. It is assumed that restoration of a more symmetrical weight distribution is associated with improved postural stability. *Objective*. To determine to what extent WBA is associated with postural instability in people after stroke. *Methods*. Electronic databases were searched (Cochrane, MEDLINE, EMBASE, and CINAHL) until March 2012. *Main Eligibility Criteria*. (1) Participants were people after stroke. (2) The association between WBA and postural stability was reported. Quality of reporting was assessed with the STROBE checklist and a related tool for reporting of confounding. *Results*. Nine observational studies met all criteria. Greater spontaneous WBA was associated with higher center of pressure (COP) velocity and with poorer synchronization of COP trajectories between the legs (two and one studies, resp.). Evidence for associations between WBA and performance on clinical balance tests or falls was weak. *Conclusion*. Greater WBA after stroke was associated with increased postural sway, but the current literature does not provide evidence for a causal relationship. Further studies should investigate whether reducing WBA would improve postural stability.

## 1. Introduction

Of all possible sensorimotor consequences of stroke, impaired postural stability probably has the greatest impact on gait and independency in activities of daily living (ADL) [1, 2]. Indeed, to achieve improvement in walking capacity or ADL, improvement in standing balance is more important than improvement in leg strength [3]. Moreover, balance impairments are a major risk factor for falls [4]. Falling is a very common complication after stroke, with as many as 50% to 70% of the people who return home from the hospital or rehabilitation center experiencing falls [5]. These falls can have severe consequences such as hip fractures and decreased physical activity due to fear of falling [4]. Improvement of postural stability is, therefore, an important goal in stroke rehabilitation [2, 3]. 

Another common consequence of stroke is weight-bearing asymmetry (WBA). During quiet stance a substantial amount of WBA in favor of the nonparetic leg is commonly observed [6–8]. Although asymmetry significantly improves during the first weeks of rehabilitation, some degree of WBA persists (on average 10% more weight being borne on the nonparetic leg) [9–11]. This asymmetry increases during dual-task performance [11], suggesting that weight-bearing on the paretic leg is not easily performed automatically. 

Historically, training of weight-bearing symmetry has been a major focus in balance rehabilitation after stroke. Facilitation of normal movement patterns and symmetry in weight-bearing are among the key principles of the Bobath concept and related Neurodevelopmental Treatment (NDT) [12]. These training approaches implicitly assume that restoration of a more symmetrical weight distribution is associated with improved postural stability [13]. In the same vein, several specific interventions, such as compelled weight-bearing through shoe lifts and biofeedback training, have been developed to re-establish weight-bearing symmetry in people after stroke [6, 14–17]. Weight-bearing asymmetry was reduced by these interventions; however, whether this was associated with improvement of postural control was not reported. 

In order to better understand the clinical meaning of weight-bearing asymmetry for balance rehabilitation after stroke, it is important to know how WBA and postural stability are correlated. The aim of this systematic review was, therefore, to determine to what extent WBA is associated with postural instability in people after stroke. For this review a broad definition of postural stability was chosen: “the ability of a person to maintain, achieve or restore balance, or to avoid falling” [18].

## 2. Methods

A systematic review was conducted according to the PRISMA statement where applicable [19]. It should be noted that the PRISMA statement was designed for systematic reviews of intervention studies and, therefore, several points could not be addressed in this study.

### 2.1. Eligibility Criteria

To be included studies had to meet the following eligibility criteria:

1. publication type: there were no restrictions on type of study; however, only papers published in peer-reviewed journals were included;
2. participants: subjects had to have sustained a stroke, regardless of stroke type or poststroke duration;
3. outcome measures: the study contained data on the association between WBA and (any measure of) postural stability. Intervention studies aimed at restoring weight-bearing symmetry were only considered for inclusion if they reported on the association between WBA and postural stability. Measures for WBA were accepted if they quantified the differences in weight borne on either leg;
4. language: the study was written in the English, German, or Dutch language.

### 2.2. Information Sources

Studies were identified by searching the following electronic databases: Cochrane, MEDLINE, EMBASE, and CINAHL. The search was conducted by the first author (J. Kamphuis) in March 2012. There were no restrictions regarding publication date. In addition, reference lists of the included studies were screened for potentially eligible studies.

### 2.3. Search Strategy

The following MESH terms were used for the search in MEDLINE:

1. “Stroke,” “Brain Infarction”, “Cerebrovascular Disorders”, “Brain Ischemia”, “Paresis”, “Hemiplegia”, “Intracranial Hemorrhages”, “Intracranial Embolism and Thrombosis”
2. “Weight-Bearing”
3. “Postural Balance”, “Posture”

Details on the full search strategy in MEDLINE are available in Appendix A. For the other databases the same terms were used where possible.

### 2.4. Study Selection

Study selection was initially performed by the first author (J. Kamphuis) and then checked by the second author (D. de Kam). First, the titles and abstracts of the publications retrieved by electronic searching were screened. Second, potentially eligible studies were retrieved full text before definitive inclusion. In the case of disagreement between the two authors, the last author (V. Weerdesteyn) was consulted to decide whether a study was included.

### 2.5. Data Extraction

Data were extracted from the studies by J. Kamphuis and then checked by D. de Kam. The extracted data were discussed until consensus was reached. In the case of disagreement the last author (V. Weerdesteyn) was consulted. No specific form was used for data extraction; however, the outcomes to be extracted were defined a priori. The following information was extracted from each study:

1. author and year of publication,
2. study design,
3. number of participants,
4. demographic participant characteristics: age and time after stroke,
5. baseline measures of stroke severity,
6. baseline measures of clinical balance and/or ambulatory performance,
7. dependent measures of postural stability,
8. statistics for association between WBA and postural stability.

### 2.6. Definition of Measures of Postural Stability

Of each study we summarized the reported results regarding the association between postural stability and WBA, regardless whether this association was the focus of the original study or not (Table 1). Because a variety of measures of postural stability have been reported in the literature, they were categorized by the methods of data collection:

1. quiet standing posturography: postural sway measured with force plates, usually expressed as center of pressure (COP) amplitude or COP velocity,
2. dynamic posturography: postural responses to balance perturbations. These responses are measured quantitatively in terms of COP excursions, muscle onset latencies, kinematic parameters etcetera;
3. clinical balance tests: these tests are typically used by clinicians to evaluate balance performance during functional tasks such as reaching or one-legged standing;
4. falls in daily life: the ultimate consequence of postural instability is falling. Therefore, the number of falls in daily life was also considered as a measure of postural stability.

Results regarding the association between postural stability and WBA were presented as

1. positive: if greater WBA was associated with more postural instability,
2. negative: if greater WBA was associated with less postural instability.

### 2.7. Quality of Reporting in Individual Studies

In a recent systematic review it was concluded that there is currently no quality assessment tool available for observational studies [20]. We, therefore, used a checklist for authors of observational studies that was developed by a group of expert methodologists, researchers, and editors: “Strengthening the Reporting of Observational Studies in Epidemiology” (STROBE) [21] to globally assess the reporting quality of the included studies. Although this checklist was not intended as a quality assessment tool, the items were considered relevant by a group of experts in the field. Scoring definitions were derived from the STROBE “Explanation and Elaboration” document [22] and are available in Table 4.

The risk of bias of the included studies was assessed with an instrument developed by Groenwold and coworkers [23]. This tool was specifically designed to rate the report of confounding bias in observational studies based on the STROBE statement.

All included studies were scored by J. Kamphuis and D. de Kam independently. Discrepancies were discussed between the two authors until consensus was reached. If necessary, a third assessor (V. Weerdesteyn) was consulted.

## 3. Results

### 3.1. Study Selection

**Figure 1:**
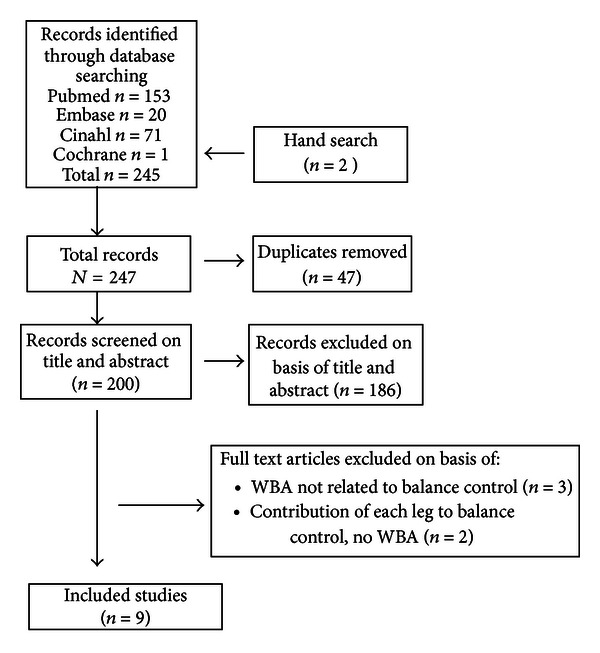
Flow chart of systematic review of weight-bearing asymmetry (WBA) related to postural control.

A total of 247 articles were retrieved by electronic searching. After screening of titles and abstracts 14 articles were selected. Finally, after full text reading of these 14 articles, nine studies met the eligibility criteria (Figure 1).

### 3.2. Study Characteristics

Characteristics of the nine included studies are reported in Table 1. Seven were cross-sectional studies and two were longitudinal cohort studies. However, one longitudinal study analyzed the association between WBA and postural stability cross-sectionally and was, therefore, regarded as a cross-sectional study [24]. Seven of the nine included papers were originally designed to determine the association between WBA and a measure of postural stability [8, 10, 25–29]. In the other two studies this association was reported as a secondary analysis or it could be extracted from a larger regression model [24, 30]. 

Descriptive data on balance and gait capacities of the subjects was provided in all studies except two [8, 27]. These functional capacities varied greatly between the studies, from a Berg Balance Scale (BBS) of 4 points [10] at the lower end, up to a maximum BBS score of 56 [10, 29, 30] or being able to walk independently without supervision (Functional Ambulation Categories 4-5) [24, 28, 29]. As for measures of postural stability, quiet standing posturography was done in five studies [10, 24, 25, 28, 30], dynamic posturography in two studies [26, 29], clinical balance tests in two studies [27, 30], and number of falls was recorded in two studies [8, 30]. The study by Mansfield et al. [30] assessed postural control using all categories except dynamic posturography.

### 3.3. Quality of Reporting in Individual Studies

The quality of reporting of individual studies according to the STROBE criteria and the scores on the risk-of-bias checklist are shown in Tables 2 and 3. 

The criteria for the reporting of study rationale, objectives, outcome variables, data analysis, and study results were satisfied in at least seven of the nine studies. Criteria for reporting recruitment sites and methods of participant selection were only sufficiently reported in five and six studies, respectively. In addition, none of the studies clearly justified their sample size. The generalizability of the results was discussed in only five of the nine studies.

For the reporting of confounding bias only one study [30] scored seven points (out of 8). The remaining studies had a total score of four points or less (Table 3). Three studies applied a method to correct for potential confounders in their analysis [24, 26, 30]; however, only two studies justified their choice for the selection of potential confounders [25, 30]. The possibility of unobserved confounding was discussed in five of the studies [8, 25, 27, 29, 30].

### 3.4. Results of Individual Studies

Measures of postural stability varied considerably among the studies, even within each of the four categories. Therefore, meta-analysis of the data was not considered appropriate. 

#### 3.4.1. Definitions of Weight-Bearing Asymmetry

In most studies WBA was expressed as a function of body weight (BW) [10, 26, 28–30]. Most of the studies also considered the direction of WBA (towards the affected or unaffected side) [8, 10, 25–28], whereas others only calculated the absolute degree of WBA [24, 30]. In the remaining studies WBA was calculated as the loading ratio between the affected and unaffected leg [27], the difference between loading on the affected versus unaffected leg divided by 50% of body weight [25], or the deviation of the COP from the midline between the legs in the frontal plane [8, 24]. Due to these differences it was not possible to compare absolute effect sizes between different studies. 

#### 3.4.2. Spontaneous and Imposed Weight-Bearing Asymmetry

In most studies participants were allowed to self-select their weight distribution (spontaneous WBA). In one study participants were instructed to stand as symmetrically as possible [24]. In the experiment of Marigold et al. (2004), three different conditions of WBA were imposed within the same subjects [26].

#### 3.4.3. Associations between Weight-Bearing Asymmetry and Quiet Standing Posturography

The correlation between spontaneous WBA and quiet standing balance was determined in four studies [10, 25, 28, 30]. In another study, the association between imposed weight-bearing symmetry and postural sway was reported [24]. 

Two cross-sectional studies with a total sample of 73 participants in the chronic phase after stroke analyzed the correlation of WBA with measures of postural sway [25, 28]. Both studies reported that greater WBA was associated with more postural sway. These associations were most evident for COP velocity; however, the explained variance was moderate (*R* ^2^ = 0.18–0.25). The study by Roerdink et al. did not find a significant association between WBA and total sway area [24]. In this study, however, participants were instructed to stand as symmetrically as possible. 

In the two studies by Mansfield et al. [10, 30] the correlation was examined between WBA and between-limb synchronization of COP trajectories. Synchronization was expressed as the cross-correlation coefficient between the individual COP trajectories under the left and right foot. High correlation coefficients indicated that regulatory activity was synchronized between the paretic and nonparetic legs and was interpreted as the two legs working together to control posture. In both studies, WBA was associated with low between-limb synchronization, but this was significant in only one of the studies (*R* ^2^ = 0.19–0.23) [10]. 

#### 3.4.4. Associations between Weight-Bearing Asymmetry and Dynamic Posturography

The correlation between WBA and dynamic posturography outcomes was investigated in two studies [26, 29]. These studies included a total of 18 individuals with stroke. Participants stood on a platform and were instructed to maintain their balance, while random platform movements (discrete [26] or continuous [29]) were applied in forward and backward directions. The study of Marigold et al. [26] used a within-subjects design with three different stance conditions of imposed WBA: increased weight-bearing load, decreased weight-bearing load, and self-selected stance. This study found no significant differences in muscle onset latency or amplitude of muscle activity between the three load conditions in individuals with stroke, except for an increase in gastrocnemius amplitudes with more weight-bearing in both the paretic and nonparetic limbs. In contrast, in healthy controls increased loading shortened the onset latencies of gastrocnemius and increased the amplitude of tibialis anterior and gastrocnemius responses. 

In the study by van Asseldonk et al. [29] the “dynamic balance contribution” of each leg to postural control was assessed by comparing the ankle joint torques of the paretic with the nonparetic limb in individuals with stroke and healthy subjects. A key finding of this study was that the contribution of the paretic leg to balance was much smaller (on average about 10–20%) than its contribution to weight-bearing (on average about 40–45%). More weight-bearing on the paretic leg tended to be associated with a larger contribution to postural control; however, this correlation was not significant (*R* ^2^ = 0.26, *P* = 0.24). Conversely, in healthy subjects adopting an asymmetrical posture, the contribution of each leg to postural control equaled its contribution to weight-bearing. 

#### 3.4.5. Associations between Weight-Bearing Asymmetry and Clinical Balance Performance Tests or Falls in Daily Life

The correlation between WBA and clinical balance performance tests was examined in two studies [27, 30]. Although Mansfield et al. [30] observed a significant association of greater WBA with poorer BBS scores, the explained variance was very low (5-6%). It must be mentioned, however, that their study was not designed to specifically investigate this relationship and that the reported association was derived from a larger regression model. In the study by Pereira et al. [27] no significant correlations between Functional Reach and WBA were found for the total number of 14 participants. However, when only the patients with WBA towards the nonparetic leg were considered (*n* = 10), greater WBA was significantly associated with better Functional Reach scores (*R* ^2^ = 0.49). 

The association between WBA and falls was measured in two studies [8, 30]. In the study of Sackley [8] falls were recorded both prospectively and retrospectively from patient charts and by interviews. Mansfield and coworkers retrospectively collected data on both falls and postural stability from patient charts [30]. Both studies did not find significant associations. Again, the association reported by Mansfield and coworkers [30] was derived from a regression analysis that was conducted to answer a different research question. 

## 4. Discussion

The purpose of this review was to determine the association between WBA and postural stability in people after stroke. Nine observational studies were found, of which seven were originally designed to investigate this relationship. Measures of postural stability were very diverse among the included studies and were, therefore, categorized according to the methods used for data collection. For static posturography the general trend was that greater WBA was associated with larger COP velocities. The strengths of the associations were, however, moderate at best. 

### 4.1. Static and Dynamic Posturography

In two studies that recorded overall COP excursions (i.e., of the paretic and nonparetic leg combined) greater WBA was associated with increased postural sway [25, 28]. Interestingly, the observed associations appeared to be stronger for measures of COP velocity than COP amplitude. COP velocity measures are not only more reliable than measures of COP amplitude [31, 32], but also sensitive to changes in the frequency of regulation. This is important because particularly the higher frequencies within the COP fluctuations reflect the stabilizing ankle torques. Hence, the finding that greater WBA was more strongly associated with greater COP velocities than COP amplitudes suggests that more regulatory activity was applied for maintaining upright stability when a more asymmetrical weight distribution was adopted. 

Several other studies included in this review measured the individual paretic and nonparetic COP trajectories, which enabled the researchers to determine the regulatory activity of each leg separately [10, 24, 29, 30]. In healthy people, adopting an asymmetric weight distribution results in increased regulatory activity (i.e., COP velocities) under the most loaded leg, thereby increasing its relative contribution to postural control [33]. In the studies by Roerdink et al. [24] and van Asseldonk et al. [29] the regulatory activity under the paretic leg was found to be substantially lower compared to the nonparetic leg. In the study by van Asseldonk et al. [29] the explained variance (*R* ^2^ = 0.26) between WBA and the contribution of the paretic leg to postural control was in the same order of magnitude as the values reported for overall COP velocity (*R* ^2^ = 0.18–0.25), but the correlation did not reach significance, possibly due to the limited sample size (*n* = 8). Nevertheless, the average contribution of the paretic leg amounted to as little as 10–20% while bearing 40–45% of the body weight, whereas in healthy subjects the weight borne on a leg equaled its contribution to postural control when adopting an asymmetric position [29]. 

The study by Mansfield and coworkers [10] provided further insight into the temporal aspects of corrective actions under the paretic and nonparetic legs as assessed by cross-correlation of the individual COP trajectories. Greater WBA was associated with poorer synchronization (i.e., lower cross-correlation coefficients) of COP trajectories (*R* ^2^ = 0.19–0.23) [10], which indicated that the paretic and nonparetic legs less adequately worked together in controlling balance when adopting a more asymmetric weight distribution. It was shown that the synchronization of left and right COP trajectories was almost perfect (i.e., cross-correlation coefficients close to 1.0) in healthy persons, but it was not investigated whether and how this would be affected by WBA.

The effect of WBA on the timing of balance correcting responses after support-surface translations was investigated by Marigold et al. [26]. This was the only study using a within-subjects design. In contrast to healthy controls, people with stroke demonstrated largely absent modulation of response latencies and amplitudes with different degrees of WBA. In other words, imposing weight-bearing symmetry resulted in only minimal improvement in the corrective postural responses of the paretic leg. Overall, the muscular responses of the patients were delayed compared to the healthy controls. These findings suggest that WBA is not the primary cause of the reduced postural stability after stroke. This suggestion is supported by several other observations. First, the degree of postural instability after stroke greatly exceeds the effect of adopting different degrees of WBA on postural stability in healthy persons (within the ranges that are commonly observed after stroke) [11, 33]. Second, the disproportionately low contribution of the paretic leg to postural control (relative to the amount of weight borne on the paretic leg) as reported by van Asseldonk et al. [29] also argues against a major role for WBA in the causation of postural instability after stroke.

Alternatively, we suggest that WBA after stroke may be regarded as a compensatory strategy to enhance the kinetic contribution of the nonparetic leg to balance. Several findings support this notion. First, it has been found in a longitudinal cohort study that, although WBA decreased within the first weeks after stroke and postural stability improved over a period of at least another two months, the regulatory activity of the paretic leg (expressed as COP velocity) did not improve [11]. In the same vein, the contribution of the paretic leg to postural control was found to be as little as 10–20% despite good recovery of ambulatory capacity (FAC 4-5) and balance performance (BBS > 45) in the chronic phase after stroke [29]. These findings suggest that improvement of postural stability after stroke is primarily driven by compensatory strategies rather than by the restoration of motor control of the affected leg. Second, Roerdink and coworkers found that both WBA and a reduced contribution of the paretic leg to postural control were most evident in individuals with a lack of selective muscle control of the paretic leg [24]. These patients may have used WBA to enhance the kinetic contribution of the nonparetic leg to balance. 

Compensation for decreased regulatory activity by the paretic leg as a possible explanation for persistent WBA after stroke does, however, not preclude the possibility of other underlying mechanisms. For instance, recent evidence suggests that misperception of the postural and visual vertical after stroke may also contribute to WBA while standing, particularly in patients with visuospatial hemineglect [9, 34]. From this perspective, WBA may be regarded as a primary impairment rather than a secondary compensation.

Although WBA towards the nonparetic leg may be a beneficial compensation for the reduced regulatory activity by the paretic leg while quiet standing, it remains to be investigated to what extent WBA is advantageous for dynamic postural stability, such as when stepping to recover balance after an external perturbation. In a recent study it was found that after stroke WBA towards the nonaffected side was associated with an increased likelihood of stepping with the paretic leg in response to a forward perturbation [35]. It was suggested that this strategy may be less effective to restore balance than to step with the nonparetic leg. Further research should, therefore, shed light on the effects of different degrees of WBA on a variety of postural tasks.

### 4.2. Clinical Balance Performance

Whereas static and dynamic posturography provide information on underlying postural control mechanisms, clinical balance tests such as the Berg Balance Scale and the Functional Reach test evaluate functional capacities. They allow for adaptive strategies to compensate for the underlying impairments to accomplish the required task. The results of the two studies that reported on the correlation between WBA and clinical balance tests do not provide conclusive evidence for the magnitude or direction of a possible association. First, the association between greater WBA and lower BBS scores was indirectly derived from a larger regression model as reported in the study by Mansfield et al. [30], which may explain the relatively small explained variance. As this study was not originally designed to investigate this association and the regression model contained other measures of postural control as well, it is difficult to draw any conclusions from this finding.

The study by Pereira et al. (2010) was the only one that reported greater WBA to be associated with better postural stability as measured with the Functional Reach test, but this only concerned the individuals bearing more weight on the nonparetic leg [27]. This finding suggests that WBA may be an effective compensatory strategy during functional (reach) tasks. Yet, further research is needed to confirm this notion.

### 4.3. Falls in Daily Life

The ultimate consequence of postural instability is falling, which was therefore regarded as a measure of postural stability as well. Although the two studies that reported on the association between WBA and falls in daily life found no significant correlations, these results should be interpreted cautiously. First, the study by Mansfield et al. was not designed to specifically assess this association [30]. Second, in the study by Sackley, the fall data collection did not comply to the currently accepted methods, which may have influenced the results [8].

### 4.4. Limitations

The number of studies reporting on the association between WBA and postural stability was limited (*N* = 9), with only seven studies being originally designed to investigate this association. Another limitation was the variety of balance measures that assessed different aspects of postural control, which limited the possibility to compare outcomes. This problem was only partially overcome by categorization of the balance measures. Besides differences in measures of postural stability, study populations varied greatly in terms of poststroke duration and functional capacities. Although most studies only considered participants with unilateral stroke, two studies also included patients with bilateral stroke [8, 30]. Furthermore, two studies [24, 30] did not consider the direction of WBA (towards the paretic or nonparetic side), while the study by Pereira et al. [27] demonstrated that associations between WBA and postural stability may be dependent on this direction. 

The reporting of confounding bias was poor in all studies except one [30]. In only three studies a method to correct for confounders was applied [24, 26, 30]. One important confounder that was often not accounted for was stroke severity. Postural instability and WBA are probably both related to stroke severity [7, 9, 11, 34, 36, 37] and, consequently, these phenomena will be strongly correlated. Yet, to better understand the causal relationship between WBA and postural stability after stroke, the effect of different degrees of imposed WBA on postural control should be investigated within the same group of patients. 

## 5. Conclusion

Overall, the studies included in this review suggest that WBA after stroke is associated with increased postural sway as well as with poorer between-limb synchronization of COP trajectories. These associations were obtained from cross-sectional studies, which do not provide insight into causal relationships. Yet, the one study that imposed different degrees of weight-bearing symmetry does not provide support for WBA being a main cause of postural instability in people with stroke. Whether adopting an asymmetric weight distribution (in favour of the nonparetic leg) is detrimental or beneficial for both static and dynamic postural stability after stroke remains to be investigated for various degrees of stroke severity (in particular the severity of paresis).

## Figures and Tables

**Table 1:** Study characteristics.

Author (year)	Study design	Number of participants with stroke	Participant characteristics (1) Age (years) (2) Time after stroke	Baseline measures of stroke severity	Baseline measures of clinical balance /ambulatory performance	Category of balance measure	Dependent measures of postural stability	Statistics for association between WBA and postural stability
Van Asseldonk et al. (2006) [29]	Cross-sectional	*N* = 8	(1) 59.9 (8.3) (2) 42.4 (17.1) months	MI 51.5 (16.1)	FAC (4-5) BBS 49 (7.6) TUG 21.8 (11.9) TBT (3–4)	DP	Contribution of paretic leg to balance control^a^	↑ *R* ^2^ = 0.26

Mansfield et al. (2011) [10]	Cross-sectional	*N* = 33	(1) 61 (14) (2) 133 (194) days	NIHSS 4.6 (4.1) ChMcS-leg 4.6 (1.3) ChMcS-foot 4.2 (1.6)	BBS 37.9 (15.6)	QS	COP amplitude synchronization^b^	**↑ ** **R** ^2^ ** = 0.19–0.23**

Mansfield et al. (2012) [30]	Cross-sectional Retrospective chart Review	*N* = 100	(1) 66.9 (14.9) (2) 22.9 (23.5) days	NIHSS 3.5 (3.0) ChMcS-leg 4.8 (1.3) ChMcS-foot 4.5 (1.4)	BBS 36.9 (14.6)	QS CB FA	COP amplitude synchronization^b^ BBS Faller status	↑ *R* ^2^ = 0.032–0.053 **↑ ** **R** ^2^ ** = 0.049**–**0.057** **Multiple linear regression** *P* = 0.060 Multiple logistic regression

Marigold et al. (2004) [26]	Cross-sectional	*N* = 10	(1) 61.3 (8.9) (2) 4.1 (2.9) years	Not reported	BBS 44.9 (8.3)	DP	TA amplitude MG amplitude TA onset latencies MG onset latencies	F_2,18_ = 1.51_(p)_, 0.67_(np)_ **↑ ** **F**_2,18_ ** = **6,14_(**p**)_,** **3,78_(**np**)_ **Anova** F_2,18_ = 0.15_(p)_, 0.78_(np)_ F_2,18_ = 0.19_(p)_, 0.70_(np)_

Marigold and Eng (2006) [25]	Cross sectional	*N* = 28	(1) 62.1 (8.6) (2) 4.1 (2.7) years	ChMcS-leg 4 (3-5) ChMcS-foot 5 (4–6)	BBS 46.2 (42.4–48.6) IQR	QS	COP amplitude (AP) COP velocity (AP) COP amplitude (ML) COP velocity (ML)	↑ *R* ^2^ = 0.0049 ↑ *R* ^2^ = 0.01 ↑ *R* ^2^ = 0.044 **↑ ** **R** ^2^ ** = 0.22**

Pereira et al. (2010) [27]	Cross-sectional	*N* = 14	(1) 65 (10) (2) 29 (23) months	Not reported	Not reported	CB	Functional Reach	NS total group ↓*R* ^2^ = 0.10

Peurala et al. (2007) [28]	Cross-sectional	*N* = 45	(1) Left hemi 53.4 (8) Right hemi 55.1 (8) (2) Left hemi 3.1 (4.8) years Right hemi 2.8 (2.8) years	FIM Left hemi 103.4 (10) Right hemi 101.1 (13)	FAC (2–5)	QS	COP velocity (AP) COP velocity (ML) Power peak magnitude (AP)^c^ Power peak magnitude (ML)^c^	**↑ ** **R** ^2^ ** = 0.18,** **↑ ** **R** ^2^ ** = 0.25** **↑ ** **R** ^2^ ** = 0.09–0.14** **↑ ** **R** ^2^ ** = 0.12–0.20**

Roerdink et al. (2009) [24]	Cross-sectional*	*N* = 33	(1) 61.2 (13.0) (2) 9.8 (5.4) weeks	BFM II–VI	FAC 1–4	QS	Sway area^d^	↑ *R* ^2^ = 0.073

Sackley (1991) [8]	Longitudinal cohort	*N* = 92	(1) 63.3 (21–87) (2) 11.3 (5) weeks	Not reported	Not reported	FA	Number of falls	NS

Values for descriptive data represent mean (sd) unless stated otherwise. Statistics represent *R* ^2^ as calculated from bivariate correlations unless stated otherwise. Significant associations are presented in bold. ↑: greater WBA is associated with more postural instability, ↓: greater WBA is associated with less postural instability AP: anteroposterior, BBS: Berg Balance Scale, BFM: Brunnstrom Fugl-Meyer assessment, CB: clinical balance test, ChMcS: Chedoke-McMaster Stroke assessment scores, COP: center of pressure, DP: dynamic posturography, FA: falls in daily life, FAC: Functional Ambulation Categories, FIM: Functional Independence Measure, FR: Functional Reach test, IQR: interquartile range, MG: medial gastrocnemius, MI: Motricity Index, ML: mediolateral, *N*: number of stroke participants, NIHSS: National Institutes of Health Stroke Scale, NP: non-paretic, NS: no significant association with WBA, p: paretic, QS: quiet standing posturography, TA: tibialis anterior, TBT: Timed Balance Test, TUG: Timed Up and Go test, WBA: weight-bearing asymmetry. *Longitudinal study, but correlations between WBA and postural control were analyzed cross-sectionally. ^a^% contribution of the paretic leg to total amount of generated corrective torque. ^b^Calculated by cross-correlating COP amplitude time series, ^c^power spectral density functions, ^d^total area covered by the COP trajectory.

**Table 2:** Reporting quality of individual studies according to the STROBE criteria.

		Van Asseldonk et al. (2006) [29]	Mansfield et al. (2011) [10]	Mansfield et al. (2012) [30]	Marigold et al. (2004) [26]	Marigold and Eng (2006) [25]	Pereira et al. (2010) [27]	Peurala et al. (2007) [28]	Roerdink et al. (2009) [24]	Sackley (1991) [8]	Proportion of studies that satisfied criteria
	1a	0	0	1	0	0	0	0	1	0	2/9 (22%)
	1b	1	1	1	1	1	1	1	1	1	9/9 (100%)
Background/rationale	2	1	1	1	1	1	1	0	1	1	8/9 (89%)
Objectives	3	1	1	1	1	1	1	1	1	1	9/9 (100%)
Study design	4	0	0	0	0	0	0	0	1*	0	1/9 (11%)
Setting	5	0	1	1	0	0	1	0	1*	1	5/9 (56%)
Participants	6a	0	1	1	0	0	1	1	1*	1	6/9 (67%)
	6b	1	0	NA	0	0	NA	0	NA	NA	1/5 (20%)
Variables	7	1	1	1	1	1	1	1	1	1	9/9 (100%)
Data sources/measurement	8	1	1	1	1	1	1	1	1	0	8/9 (89%)
Bias	9	0	0	1	1	1	0	0	1	0	4/9 (44%)
Study size	10	0	0	0	0	0	0	0	0	0	0/9 (0%)
Quantitative variables	11	1	1	1	1	1	1	1	0	0	7/9 (78%)
Statistical methods	12a	1	1	1	1	1	1	1	1	1	9/9 (100%)
	12b	NA	NA	NA	NA	1	1	NA	1	NA	3/3 (100%)
	12c	1	1	0	NA	NA	NA	NA	0	1	3/5 (60%)
	12d	NA	NA	NA	NA	NA	NA	NA	1*	1	2/2 (100%)
	12e	0	0	0	0	0	0	0	0	0	0/9 (0%)
Participants	13a	0	0	0	0	0	0	0	0	0	0/9 (0%)
	13b	NA	1	0	NA	NA	NA	NA	0*	1	2/4 (50%)
	13c	NA	NA	NA	NA	NA	NA	NA	0	0	0/2 (0%)
Descriptive data	14a	1	1	1	1	1	0	1	1*	1	8/9 (89%)
	14b	1	1	0	1	1	1	1	0	1	7/9 (78%)
	14c	NA	NA	0	NA	NA	NA	NA	1*	1	2/3 (67%)
Outcome data	15	1	1	1	1	1	1	1	1	1	9/9 (100%)
Main results	16a	1	1	1	1	1	1	1	1	0	8/9 (89%)
	16b	NA	NA	NA	NA	NA	NA	1	NA	NA	1/1 (100%)
	16c	NA	NA	0	NA	NA	NA	NA	NA	NA	0/1 (0%)
Other analysis	17	NA	NA	NA	NA	1	0	NA	1	1	3/4 (75%)
Key results	18	1	1	1	0	0	0	1	1	0	5/9 (56%)
Limitations	19	1	0	1	1	1	0	0	1	1	6/9 (67%)
Interpretation	20	1	0	1	1	1	0	0	1	1	6/9 (67%)
Generalizability	21	1	1	1	0	0	1	0	1	0	5/9 (56%)
Funding	22	0	1	1	0	0	0	1	0	0	3/9 (33%)

Total score		17/26	18/27	19/28	14/25	16/27	14/26	14/26	22/31	17/30	

Percentage		65%	67%	68%	56%	59%	54%	54%	71%	57%	

*Score is based on the original study of de Haart et al. 2004 [11].

**Table 3:** Reporting of confounding bias in individual studies [23].

	Van Asseldonk et al. (2006) [29]	Mansfield et al. (2011) [10]	Mansfield et al. (2012) [30]	Marigold et al. (2004) [26]	Marigold and Eng (2006) [25]	Pereira et al. (2010) [27]	Peurala et al. (2007) [28]	Roerdink et al. (2009) [24]	Sackley (1991) [8]	Number of studies satisfied criteria
(1) Reporting of reasons why potential confounders are selected for analysis	0	0	1	0	1	0	0	0	0	2
(2) Reporting of reasons to include confounders in final model	0	0	1	0	1	0	0	0	0	2
(3) Reporting of characteristics of key confounders	0	0	1	0	0	0	0	0	0	1
(4) Any method used to control for confounding	0	0	1	1	0	0	0	1	0	3
(5) Reporting of both crude and adjusted effect estimate	0	0	1	0	0	0	0	0	0	1
(6) Comment on likelihood of unobserved confounding	1	0	1	0	1	1	0	0	1	5
(7) Reporting of a qualitative statement on the direction of the potential effect of unobserved confounding	0	0	1	0	1	0	0	0	0	2
(8) Sensitivity analysis used to estimate potential impact of unobserved confounding	0	0	0	0	0	0	0	0	0	0

Total score	1	0	7	1	4	1	0	1	1	

**Table 4:** STROBE criteria scoring definitions.

Part of study		Item	Score 1 if it
Title and abstract			

	1a	Indicate study design with a commonly used term in the title or in the abstract	(i) Mentions an explicit commonly used term for the study design in title or abstract
	1b	Provide in the abstract an informative and balanced summary of what was done and what was found	(i) Includes a short description of the research question, methods, results, and conclusion (ii) Provides only information presented in the article

Introduction			

Background/rationale	2	Explain the scientific background and rationale for the investigation being reported	(i) Provides important context, sets the stage for the study, and describes its focus (ii) Provides an overview on what is known on a topic and what gaps in current knowledge are addressed by the study

Objectives	3	State specific objectives, including any prespecified hypotheses	(i) States all original objectives (ii) Specifies populations, exposures, outcomes, and parameters that will be estimated

Methods			

Study design	4	Present key elements of study design early in the paper	(i) Presents study design including source population of both cases and controls point in time the sample was taken, and if applicable the follow-up period

Setting	5	Describe the setting, locations, and relevant dates, including periods of recruitment, exposure, followup, and data collection	(i) Includes recruitment sites or sources (ii) Refers to countries, towns, hospitals, or practices where the investigation took place

Participants	6a	Cohort study—give the eligibility criteria and the sources and methods of selection of participants. Describe methods of followup Case-control study—give the eligibility criteria and the sources and methods of case ascertainment and control selection. Give the rationale for the choice of cases and controls Cross-sectional study—give the eligibility criteria, and the sources and methods of selection of participants	(i) Describes all eligibility criteria and also the group from which the population was selected (ii) Details the description of methods of selection of all participants (iii) Details the of follow-up procedures if applicable
	6b	Cohort study—for matched studies, give matching criteria and number of exposed and unexposed Case-control study—for matched studies, give matching criteria and the number of controls per case	(i) Describes matching criteria, numbers of exposed and unexposed or numbers of controls per case

Variables	7	Clearly define all outcomes, exposures, predictors, potential confounders, and effect modifiers. Give diagnostic criteria, if applicable	(i) Defines all variables for and included in the analyses (ii) Clearly describes definitions of diagnostic criteria for disease outcomes, disease events, or prevalent disease (iii) Declares all candidate variables considered for statistical analysis

Data sources/measurement	8	For each variable of interest give sources of data and details of methods of assessment (measurement). Describe comparability of assessment methods if there is more than one group	(i) Refers to studies that report validity or reliability of the outcome measure OR (ii) Reports estimated validity or reliability

Bias	9	Describe any efforts to address potential sources of bias	(i) Describes what measures were taken during the conduct of the study to reduce the potential of bias (ii) If possible, discusses and estimates the direction and magnitude of bias

Study size	10	Explain how the study size was arrived at	(i) Reports formal sample size calculations if they were done, or (ii) Indicates the considerations that determined the study size

Quantitative variables	11	Explain how quantitative variables were handled in the analyses. If applicable, describe which groupings were chosen, and why	(i) For categorized variables, explains why and how data were grouped including the number of categories, the cut points, and category means or medians (ii) For data presented in tabular forms, reports counts of cases, controls, persons at risk, and so forth for each category (iii) For continuous variables, considers the nature of relation between exposure and outcome (linear, quadratic, normality, etc.).

Statistical methods	12a	Describe all statistical methods, including those used to control for confounding	(i) Describes statistical methods to enable a reader with access to the data to verify the reported results
	12b	Describe any methods used to examine subgroups and interactions	(i) Describes what methods were used for subgroup analysis (ii) Reports the way interaction effects were analyzed
	12c	Explain how missing data were addressed	(i) If applicable, reports the number of missing values for each variable of interest and for each step in the analysis (ii) Gives reason for missing values and indicates how many individuals were excluded because of missing data when describing the flow of participants through the study (iii) Describes the nature of the analyses made for missing data
	12d	Cohort study—if applicable, explain how loss to followup was addressed Case-control study—if applicable, explain how matching of cases and controls was addressed Cross-sectional study—if applicable, describe analytical methods taking account of sampling strategy	(i) Cohort: reports on the number of individuals that were lost to follow-up and how those data were treated in the analyses (ii) Case control: reports on how the matching was handled in the statistical analysis (iii) Cross-sectional: reports on methods to adjust for complex sampling strategies if they were used (e.g., clustered sampling).
	12e	Describe any sensitivity analyses	(i) Describes sensitivity analyses, for example, used to identify the degree of confounding, selection bias, or information bias required to distort an association

Results			

Participants	13a	Report the numbers of individuals at each stage of the study, for example, numbers potentially eligible, examined for eligibility, confirmed eligible, included in the study, completing follow-up, and analyzed	(i) Reports the number of individuals considered at each stage of the study from the stage of recruiting from the target population to the inclusion of participants' data in the analysis: numbers potentially eligible, examined for eligibility, confirmed eligible, included in the study, completing followup, and analyzed
	13b	Give reasons for nonparticipation at each stage	(i) If applicable, explains why participants no longer participated in the study or why they were excluded from further analysis
	13c	Consider use of a flow diagram	(i) Especially for complex studies, not applicable for small cross-sectional studies

Descriptive data	14a	Give characteristics of study participants (e.g., demographic, clinical, and social) and information on exposures and potential confounders	(i) Presents data as follows: continuous variables: mean + sd or median and percentile range categorical variables: number/proportion per category
	14b	Indicate the number of participants with missing data for each variable of interest	(i) Explicitly mentions the number of participants with missing data, OR (ii) Presents the data with sufficient details for the reader to see that all data were complete (especially for small studies)
		14c	Cohort study—summarize follow-up time (e.g., average and total amount)

Outcome data	15	Cohort study—report numbers of outcome events or summary measures over time Case-control study—report numbers in each exposure category, or summary measures of exposure Cross-sectional study—report numbers of outcome events or summary measures	(i) Presents descriptive data for exposure and outcome measures separately and not only the association between the measures

	16a	Give unadjusted estimates and, if applicable, confounder-adjusted estimates and their precision (e.g., 95% confidence interval). Make clear which confounders were adjusted for and why they were included	(i) Gives unadjusted estimates of effect size (ii) If applicable, gives adjusted estimates of effect size
Main results	16b	Report category boundaries when continuous variables were categorized	(i) Reports category boundaries when continuous variables were categorized
	16c	If relevant, consider translating estimates of relative risk into absolute risk for a meaningful time period	(i) Only for ratio measures

Other analysis	17	Report other analyses done—for example, analyses of subgroups and interactions, and sensitivity analyses	(i) Describes whether these analyses were planned in advance and which arose while analyzing

Discussion			

Key results	18	Summarize key results with reference to study objectives	(i) Begins the discussion with a short summary of the main findings of the study

Limitations	19	Discuss limitations of the study, taking into account sources of potential bias or imprecision. Discuss both direction and magnitude of any potential bias	(i) Mentions sources of bias and confounding direction of potential biases discusses any imprecision of results

Interpretation	20	Give a cautious overall interpretation of results considering objectives, limitations, multiplicity of analyses, results from similar studies, and other relevant evidence	(i) Interprets the results taking into consideration the limitations of the study or bias. (ii) Interprets the results in context of the existing external evidence from different types of studies. (iii) Puts the results in context with similar studies and how the new study affects the existing body of evidence

Generalizability	21	Discuss the generalizability (external validity) of the study results	(i) Gives sufficient information for the reader to judge the generalizability

Other information			

Funding	22	Give the source of funding and the role of the funders for the present study and, if applicable, for the original study on which the present article is based	(i) Gives the source of funding (ii) Describes the role of funders in detail

## Appendix

### A. Search Strategy MEDLINE

1. (((((((“Stroke”[Mesh]) OR “Brain Infarction”[Mesh]) OR “Cerebrovascular Disorders”[Mesh]) OR “Brain Ischemia”[Mesh]) OR “Paresis”[Mesh]) OR “Hemiplegia”[Mesh]) OR “Intracranial Hemorrhages”[Mesh]) OR “Intracranial Embolism and Thrombosis”[Mesh]
2. “cva”
3. “cerebral”
4. “stroke”
5. “cerebrovascular”
6. 1,2,3,4,5
7. (“Postural Balance”[Mesh]) OR “Posture”[Mesh]
8. “balance/physiology”
9. “balance/equilibrium”
10. “balance/coordination”
11. “balance”
12. “balance control”
13. “posture”
14. “postural”
15. 7,8,9,10,11,12,13,14
16. “weight-bearing”[Mesh]
17. “weight bearing”
18. “weight bearing/physiology”
19. “weight bearing asymmetry”
20. 16,17,18,19
21. 6, AND 15, AND 20.
